# Differences in county-level cardiovascular disease mortality rates due to damage caused by hurricane Matthew and the moderating effect of social capital: a natural experiment

**DOI:** 10.1186/s12889-022-14919-7

**Published:** 2023-01-09

**Authors:** Zachary H. McCann, Magdalena Szaflarski

**Affiliations:** 1 Department of Environmental Health, Rollins School of Public Health-Emory University, Atlanta, Georgia; 2 Department of Sociology, University of Alabama at Birmingham, Birmingham, AL United States

**Keywords:** Natural Hazard, Climate change, Cardiovascular disease, Social capital, Hurricane, Mortality, Regression adjustment, Population health

## Abstract

**Background:**

As the climate continues to warm, hurricanes will continue to increase in both severity and frequency. Hurricane damage is associated with cardiovascular events, but social capital may moderate this relationship. Social capital is a multidimensional concept with a rich theoretical tradition. Simply put, social capital refers to the social relationships and structures that provide individuals with material, financial, and emotional resources throughout their lives. Previous research has found an association between high levels of social capital and lower rates of cardiovascular (CVD) mortality. In post-disaster settings, social capital may protect against CVD mortality by improving access to life-saving resources. We examined the association between county-level hurricane damage and CVD mortality rates after Hurricane Matthew, and the moderating effect of several aspects of social capital and hurricane damage on this relationship. We hypothesized that (1) higher (vs. lower) levels of hurricane damage would be associated with increased CVD mortality rates and (2) in highly damaged counties, higher (vs. lower) levels of social capital would be associated with lower CVD mortality.

**Methods:**

Analysis used yearly (2013-2018) county-level sociodemographic and epidemiological data (*n* = 183). Sociodemographic data were compiled from federal surveys before and after Hurricane Matthew to construct, per prior literature, a social capital index based on four dimensions of social capital (sub-indices): family unity, informal civil society, institutional confidence, and collective efficacy. Epidemiological data comprised monthly CVD mortality rates constructed from monthly county-level CVD death counts from the CDC WONDER database and the US Census population estimates. Changes in CVD mortality based on level of hurricane damage were assessed using regression adjustment. We used cluster robust Poisson population average models to determine the moderating effect of social capital on CVD mortality rates in both high and low-damage counties.

**Results:**

We found that mean levels of CVD mortality increased (before and after adjustment for sociodemographic controls) in both low-damage counties (unadjusted. Mean = 2.50, 95% CI [2.41, 2.59], adjusted mean = 2.50, 95% CI [2.40, 2.72]) and high-damage counties (mean = 2.44, CI [2.29, 2.46], adj. Mean = 2.51, 95% CI [2.49, 2.84]). Among the different social capital dimensions, institutional confidence was associated with reduced initial CVD mortality in low-damage counties (unadj. IRR 1.00, 95% CI [0.90, 1.11], adj. IRR 0.91 CI [0.87, 0.94]), but its association with CVD mortality trends was null. The overall effects of social capital and its sub-indices were largely nonsignificant.

**Conclusion:**

Hurricane damage is associated with increased CVD mortality for 18 months after Hurricane Matthew. The role of social capital remains unclear. Future research should focus on improving measurement of social capital and quality of hurricane damage and CVD mortality data.

**Supplementary Information:**

The online version contains supplementary material available at 10.1186/s12889-022-14919-7.

## Introduction

Contemporary climate modeling and projections point toward a future with more extreme weather, such as catastrophic hurricanes (Saffir-Simpson Hurricane Wind Scale 4-5) [1, 2]. Epidemiological literature, meanwhile, suggests increasingly severe health consequences from such events [3, 4]. Documenting the complex interplay between natural hazards and health will help researchers and policy makers prepare for the increased incidence and prevalence of negative health outcomes as the climate continues to warm [5].

One major concern is the relationship between hurricanes and cardiovascular disease (CVD) mortality. CVD is the leading cause of death in the United States, and by 2030, about 41% of the US population will have some form of CVD [6, 7]. In post-disaster scenarios, CVD mortality increases due to shortages in the availability of food, water, medical resources, discontinuation of public services, psychological distress, and the inability to access critical information [8–12]. These sorts of post-disaster health-related concerns were present after Hurricanes Katrina and Sandy, and they were associated with increased levels of medical noncompliance, cigarette use, and difficulty accessing medication to prevent heart attacks [13, 14]. Hurricane-specific literature finds that survivors of major storms, including Hurricanes Katrina and Sandy, [14–16] are at risk for increased levels of CVD-related morbidities for up to a decade after major storm events. Although the evidence for a relationship between extreme hurricanes and CVD-related morbidities is robust, the evidence linking hurricanes and CVD mortality is limited [17]. In the current study, we fill this gap in knowledge by examining the relationship between damage caused by Hurricane Matthew and its relationship with county-level CVD mortality rates.

**Fig. 1:**
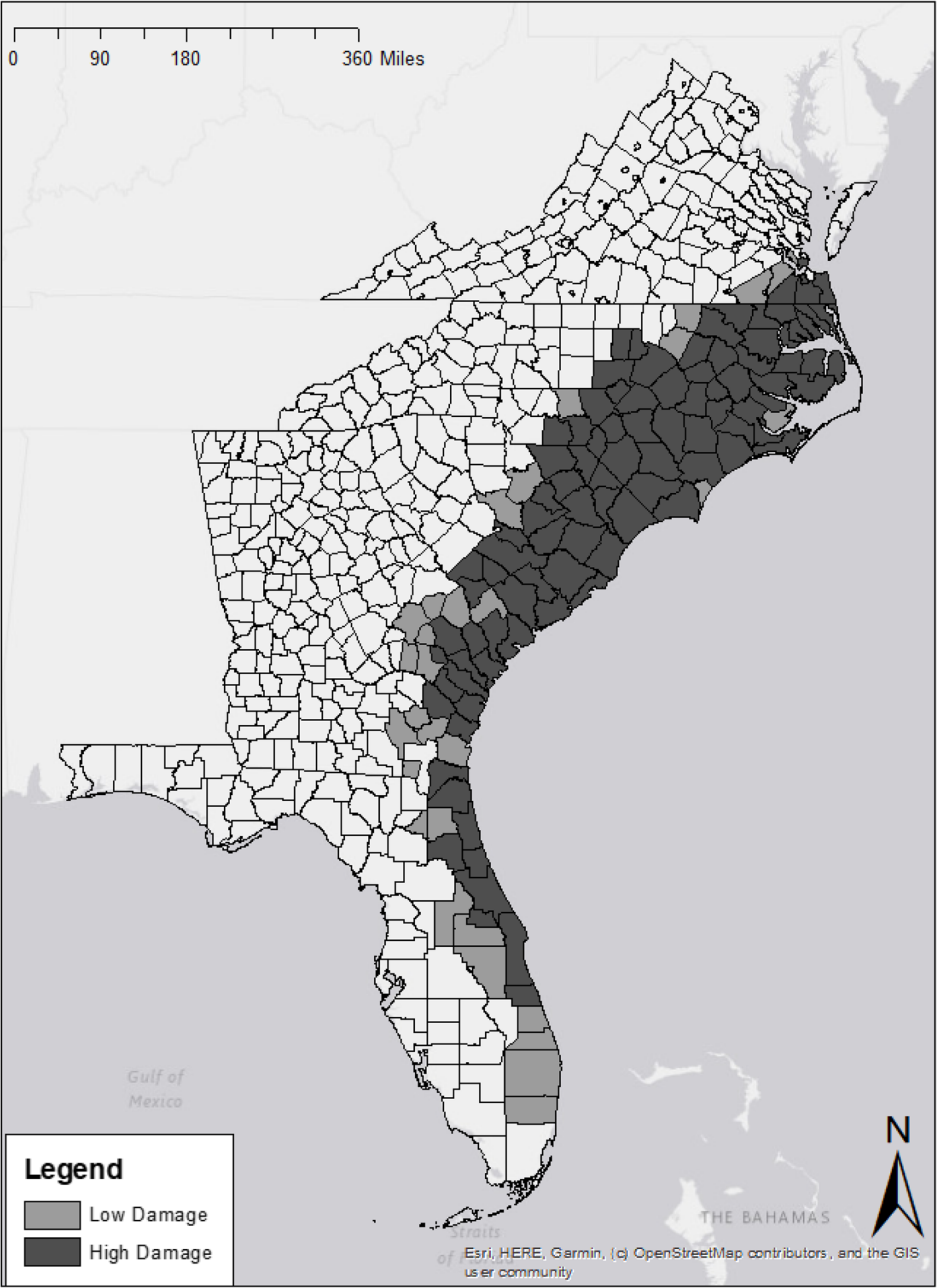
Hurricane Matthew Affected Counties and Levels of Damage. Map data sources: Base map & County boundaries [18] Level of Damage [19]

Hurricane Matthew made landfall in the United States on October 6th, 2016. The storm traveled northward along the eastern seaboard of the United States (Fig. 1) before dissipating off the coast of North Carolina on October 10th, 2016. Hurricane Matthew is notable not only for its intensity, but also for the level of rainfall that occurred. Some regions of North Carolina reported nearly 19 in. of precipitation over the course of the storm. Other states, including Florida, Georgia, and South Carolina reported over 17 in. of precipitation due to Hurricane Matthew [20]. Every state affected by Hurricane Matthew reported both coastal and river flooding. Ultimately, in the United States alone, Hurricane Matthew cost 40 lives and $10 billion in damages, making the storm one of the deadliest and economically costliest in US history [21].

Hurricane Matthew is an ideal candidate for studying the relationship between hurricane damage and CVD mortality for two primary reasons. First, most post-hurricane cardiovascular epidemiology in the United States has been conducted after Hurricanes Katrina (2005) and Sandy (2012) [17]. Studying Hurricane Matthew allowed us to confirm the relationship between hurricanes and CVD documented in previous studies, and examine the relationship between hurricane damage and CVD mortality in a different population in a new setting and allows us take advantage of more recent data. Second, Hurricane Matthew occurred over a widespread area, resulting in multiple levels of damage among otherwise similarly situated counties (Fig. 1). Subsequently, Hurricane Matthew is a prime candidate for a natural experiment that investigates the long-term effects of hurricanes on CVD mortality. The geographic range of damage caused by Hurricane Matthew also makes the storm ideal for studying how county-level social factors may alter CVD mortality outcomes after hurricanes.

One factor that may be protective against CVD declines in post-disaster settings is social capital. Social capital is generally defined as the aspects of social structure that facilitate action to achieve both individual and community goals that would not be otherwise attainable [22]. Social capital has been conceptualized in multiple works [22–26]. The conceptualization of social capital, popularized by Lin, views social capital as the availability of resources embedded in a social network as a consequence of direct and indirect social ties [25]. Others, such as Putnam, argue that social capital is a sense of trust among individuals that can result in prosocial norms and reciprocity generated by interactions in common spaces (e.g., bowling alleys, volunteer organizations, and neighborhood meetings) [23]. In contemporary scholarship, the “bonding and bridging social capital” paradigm is one of the most common ways of describing social capital [27–30]. Bonding social capital describes connections *within* a group, while bridging social capital is associated with connections *between* people and groups outside of the individual’s core networks [31]. Despite differences in the conceptualization of the exact nature of social capital, most scholars agree that social capital are the social connections that help individuals and communities generate access to otherwise unavailable resources.

There is strong evidence that social capital protects physical and mental health after hurricanes [32]. For example, social capital has been associated with lower blood pressure and reduced rates of respiratory problems after Hurricane Katrina (2005), [33] lower rates of post-traumatic stress after Hurricane Sandy (2012), [34] and lower rates of suicidal ideation after Hurricane Harvey (2018), [32]. The protective mechanisms are multi-pronged. In post-hurricane contexts, social capital should improve access to food, resources, and knowledge, ultimately reducing CVD mortality, but these relationships have not been well documented. This study is guided by a conceptual framework of associations between hurricane impacts, CVD mortality, and social capital based on the existing theory and literature (see 2.1-2.4 below).

**Fig. 2:**
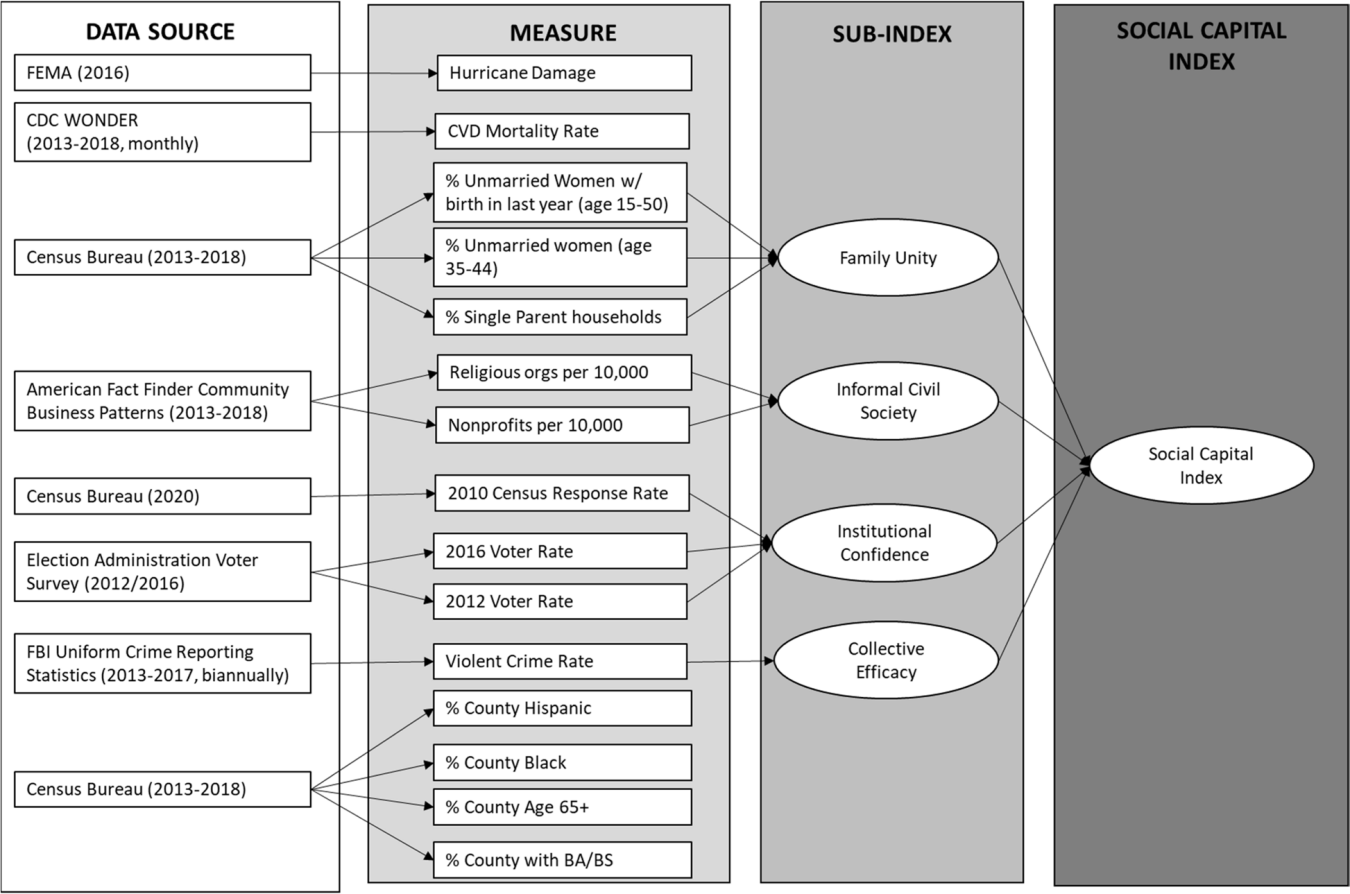
Data Sources and Division. Note: Rectangles are data sources and observed individual measures, ovals are composite indices

Conducting social capital and health research has been challenging because there is no standard measure of social capital, and some of the existing measures still warrant further assessment. A recent measure, developed by the United States Joint Economic Committee’s (JEC) Social Capital Project, is a social capital index that captures four dimensions of social capital (sub-indices) -- family unity, informal civil society, institutional confidence, and collective efficacy [35]. The JEC index uses regularly collected data to create standardized measures of multiple dimensions (sub-indices) of social capital (see Table 1 & Fig. 2). Each dimension (sub-index) addresses a unique element of social capital. Family unity intends to measure close-ties, informal-civil society measures connections across social groups, institutional confidence captures civic engagement and access to political systems, and collective efficacy is indicative of an overall sense of community trust. We selected this measure for the current study because further data are needed to establish its usefulness in health research.

**Table 1:** Data Sources, Availability, and Attrition

Variable	Source	Year(s)	Availability
Hurricane Damage	Federal Emergency Management Agency	2016	Static
CVD Mortality Count	CDC WONDER	2013-2018	Monthly
Social Capital *(sub-index)*	Multiple		
*Family Unity*			
Percent Unmarried Women (15-50) w/ Birth in the Last Year	US Census Bureau (Table: S1301)	2013-2018	Yearly
Percent Unmarried Women (35-44)	US Census Bureau (Table: B12002)	2013-2018	Yearly
Percent Single Parent Households	US Census Bureau (Table: B09002)	2013-2018	Yearly
*Institutional Civil Society*			
Number of Nonprofits County	North American Industry Classification Codes (2017)	2013-2018	Yearly
Number of Religious Organizations County	North American Industry Classification Codes (2017)	2013-2018	Yearly
*Institutional Confidence*			
2010 Census Response Rate	US Census Bureau’s “Rates for all possible geographies”	2010	Static
2012 Voter Count	Election Administration Voter Survey	2012	Static
2016 Voter Count	Election Administration Voter Survey	2016	Static
*Collective Efficacy*			
Violent Crime Rate	FBI Uniform Crime Reporting Statistics	2013-2017	Bi-annually
County Population	US Census Bureau (Table: B01003)	2013-2018	Yearly
Control Variables			
Percentage of a County Aged 65+	US Census Bureau (Table: DP05)	2013-2018	Yearly
County Income	US Census Bureau (Table: S1902)	2013-2018	Yearly
Percentage of County Residents w/ At Least BA/BS	US Census Bureau (Table: DP02)	2013-2018	Yearly
Number of County Residents Black	US Census Bureau (Table: DP02)	2013-2018	Yearly
Number of County Residents Hispanic	US Census Bureau (Table: DP02)	2013-2018	Yearly

*Note on Availability: Static = data are available once throughout the study period, bi-annually = data are available every other year, yearly = data are available once per year, and monthly = data are available once per month*

This study used data from The Federal Emergency management Agency (FEMA), Center for Disease Control and Prevention CDC, the American Fact Finder (AFF), The Census Bureau, Election Administration & Voting Survey (EAVS), and the Federal Bureau of Investigation (FBI) to examine the association between county-level hurricane damage and CVD mortality rates after Hurricane Matthew, and the moderating effect of several aspects of social capital on this relationship. We hypothesized that, by generating access to community resources that would otherwise not be available (e.g. food, potable water, medicine, energy, etc.), several aspects of social capital (assessed with the JEC-based sub-indices) would decrease rates of CVD mortality in counties damaged by Hurricane Matthew. Our research builds on previous theory and literature to further elucidate the role of social structures and processes that may reduce CVD mortality after catastrophic hurricanes, providing a pathway for future research and interventions.

## Background

### Hurricanes and CVD

The literature connecting hurricanes to CVD morbidity after Hurricanes Katrina and Sandy is extensive. Hurricane Katrina is implicated in a spike in CVD-related hospitalizations that peaked 6 days after the hurricane made landfall [36]. Evidence from hurricane Sandy aligns with the findings from Katrina. Within the elderly population (aged 76+), the risk of CVD mortality increased by a factor of 1.10 [37]. Another study in the aftermath of Hurricane Sandy found that 30 days after Sandy the risk of experiencing any CVD event decreased by a factor of .92, but the risk of mortality from cardiovascular events increased by a factor of 1.22 [16]. More recent literature has confirmed the relationships between CVD morbidity and hurricanes after Hurricane Irma (2017) [38] and across multiple tropical cyclones, [39] but additional work is needed to fully characterize the relationship between hurricanes and cardiovascular health in the United States.

### Social capital and health

Generally, social capital is thought to have a positive effect on health [26, 40–42]. A state-level study found that, relative to the lowest social capital tertile, states in the middle and highest tertiles saw a 10-11% decrease in the likelihood of residents reporting poor or fair health [43]. A study examining neighborhood social capital, characterized by contact with neighbors, was associated with improved self-rated health. In this study, each standard deviation increase in neighborhood social capital was associated with a 6% increase in the odds of neighborhood residents reporting “good or very good health,” compared to “not good health” [44]. A recent meta-analysis based on 12,778 estimates from 440 different studies confirms that social capital tends to have a modest, but consistent, positive effect on health outcomes [41].

Despite evidence of a positive relationship between social capital and health, some research suggests social capital may also transmit negative behaviors or further marginalize certain groups. This aspect of social capital has been referred to as its “dark side” [42]. Detrimental effects of social capital are most harmful to “outsiders” (e.g., residents of public housing after Hurricane Ike [45]) in otherwise cohesive communities. Others have demonstrated that different forms social capital may produce different health outcomes. Bridging social capital, or social capital generated *between* communities, [31] represented by civic, social, and volunteer groups has been associated with reduced post-hurricane poverty [46]. Bonding social capital, or the form of social capital generated by strong *within* network ties [28], represented by organizations such as religious institutions, sports clubs, labor unions, has been associated with increased post-hazard poverty [46]. Poverty, in turn, increases morbidity and mortality, and prevents people from achieving optimal health outcomes [9, 47–49].

The relationship between social capital and health after hurricanes may also be contingent on the populations and outcomes under study [45, 46]. In other words, social capital does not improve health uniformly for all people in all scenarios. Instead, social capital creates conditions that are either favorable or unfavorable for health outcomes. Aging populations, for example, are less likely to evacuate before major storms make landfall and have fewer social capital resources available in disaster situations [48, 49]. The compounding risk of age and lower social capital availability is a salient example of how social structure, social capital, and individual behaviors can work simultaneously to expose individuals and communities to differing levels of risk for CVD mortality in post-hurricane contexts.

### Social capital and CVD in post-disaster contexts

The link between natural disasters and negative CVD outcomes is clear [8–10]. Evidence also indicates that after extreme disasters, like Hurricanes Katrina and Sandy, social capital is associated with lower rates of infectious diseases, utilization of emergency departments for cardiorespiratory issues, and rates of suicidal ideation [12, 33, 50–52]. The positive association between social capital and post-hazard health outcomes is attributed to social capital improving access to life-saving resources that would not otherwise be available, including food, water, medical supplies, and shelter [37, 53]. Recent evidence suggests that these resources translate into lower CVD mortality immediately after hurricanes [33, 34]. Social capital also ameliorates, post-traumatic stress, reliance on “cheap coping” techniques, such as self-medication with alcohol and drugs, and improves access to medications and medical care after disasters [3, 53–55]. While this protective effect is expected to continue over time, evidence connecting social capital to long-term post-disaster CVD trends is limited. Furthermore, due to the difficulty of studying post-disaster populations, the mechanisms that connect severe hurricane impacts and CVD mortality are not well understood.

### Social capital conceptualization and measurement

Despite improved understanding of how social capital effects health, there are few concrete indicators of social capital. This is reflected in the tension surrounding the operationalization of social capital [54, 55]. A social capital index developed by the JEC [35] which measures social capital based on four dimensions -- *family unity, informal civil society, institutional health,* and *collective efficacy* (Fig. 2), may prove to be useful in studying the link between hurricanes and health. This measure is informed by the conceptions of social capital offered by Coleman, [22] Putnam, [23] and Small, [24] who emphasize social structure and argue that structure facilitates connections among individuals. These community-level resources facilitate social connections that work to generate access to emotional, social, material, and financial resources.

The JEC *family unity* sub-index measures family structure and stability within a county [35]. Following Coleman [22], family unity indicators are intended to point towards intergenerational network closure and relationships with close others, which are responsible for creating and transmitting intergenerational norms and beliefs. These measures are intended to capture the degree of social capital tied to relationships. The *informal civil society* sub-index captures the number of prosocial organizations per 1000 residents in a community. This sub-index reflects Small’s [39] approach to social capital. Small argues that being part of an organization, such as a church, nonprofit, or school, can yield numerous benefits, including access to institutional knowledge, resources, and relationships. Thus, this sub-index is intended to capture group participation, a behavioral element of social capital.

The third sub-index is called institutional health. In the current study, we refer to it as *institutional confidence* to avoid confusion between this sub-index and health-related outcomes. The institutional confidence sub-index measures civic participation, which is intended to measure commitment to global social norms and reflects the ability of communities to engage with local, state, and federal governments to achieve community-level goals. Thus, high levels of civic participation are indicative of structural social capital [56].

The final, fourth sub-index, *collective efficacy*, captures levels of formal and informal social control, which are associated with trust and cognitive social capital. Collective efficacy has been associated with higher levels of disaster preparedness [57]. In post-disaster contexts, collective efficacy is associated with increased levels of social support, improved access to resources, and improved mental health outcomes [58–60].

### Current study

**Fig. 3:**
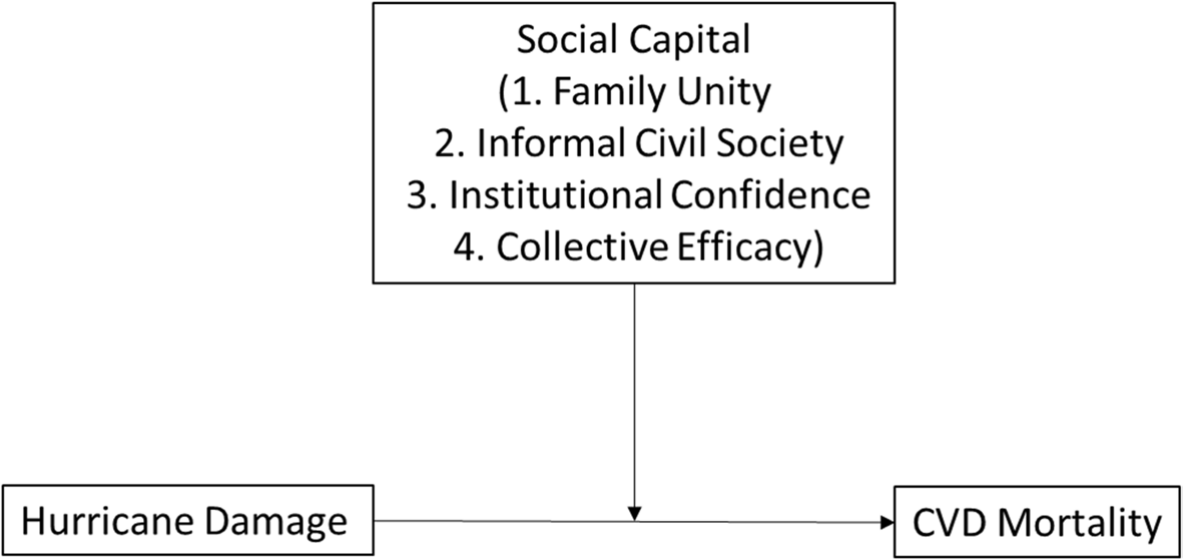
Proposed Relationship Between Hurricane Damage, CVD Mortality, and Social Capital

Considering the past theory and literature, the current study aimed to examine the following two questions: (1) Are high levels of hurricane damage associated with increased CVD mortality rates? (2) In damaged counties, does social capital reduce the rate at which CVD mortality increases? We hypothesized (Fig. 3) that (1) Higher levels of hurricane damage are associated with increased CVD mortality rates and (2) In highly damaged counties, higher levels of social capital are associated with lower CVD mortality, compared to counties with lower levels of social capital.

## Methods

### Data

To answer our research questions, we compiled publicly-available county-level data on hurricane damage severity, levels of social capital, and monthly CVD mortality rates in counties affected by Hurricane Matthew for calendar years 2013-2018 using federal data sources: CDC’s Winder-ranging Online Data for Epidemiologic Research (WONDER) system, the AFF, The Census Bureau’s table of Community Business Patterns (CBP) s, the EAVS, and the FBI’s Uniform Crime Reporting Statistics (UCR). All data analyzed were aggregated to the county level. Area-level data were combined into a single dataset, allowing us to perform an ecological analysis of the relationship between Hurricane Matthew, CVD mortality, and social capital using yearly county-level data for 2013-2018. For details on data availability and attrition, see Table 1; for a diagram on how indicators are used and how indices are constructed, see Fig. 2.

### Measurement

#### Independent variable: hurricane damage severity

We assessed hurricane severity after Hurricane Matthew, a storm that resulted in a major disaster declaration (DR, per FEMA 2018). FEMA provides information on which counties were affected by Hurricanes Matthew and the severity of hurricane damage in each county. We included all counties that received a DR (DRs 4283-4286 and 4291) due to Hurricane Matthew [19]. DRs are also used to determine which counties are eligible for public assistance only, or public and individual assistance. Following similar studies that use DRs as a metric for damage, counties receiving public and individual assistance were considered to have the most severe need for post-disaster food, shelter, water, and medical needs and were classified as “high-damage,” counties receiving public assistance only were classified as “low-damage”, and counties that did not receive any DR as “no damage” [61–64].

#### Moderating variable: social capital

Measurement of the moderating variable, social capital, is modeled after JEC [35]. We reconstructed each sub-index from the same data sources as JEC (Fig. 2).

*Family unity*. Family unity is comprised of three indicators. The first two family unity indicators, the percent of unmarried women (15-50) who had a birth in the past 12 months and the share of women ages 35-44 who are currently married (and not separated) are derived from the AFF tables S1301and B12002 [64]. The third indicator, share of single-parent households (AFF table B09002 [65]) was constructed by adding the percentage of male-headed households with children under 18 years (no mother present) and the percentage of female-headed households with children under 18 years (no father present). The result is the total number of single-parent family households.

*Informal civil society*. The informal civil society index was constructed from two indicators: the rate of nonprofits per 10,000 county residents and the rate of religious organizations per 10,000 county residents. Nonprofits and religious organizations were defined using the 2017 North American Industry Classification codes, and the total number of nonprofit or religious organizations in a county was determined using American Fact Finder’s table of Community Business Patterns [65]. In 2017, non-religious nonprofits had industry codes 8132-8139, and religious organizations were assigned industry code 8131 [66]. For each year, the number of nonprofits and religious organizations in a county were added together. County nonprofit and religious organization totals were divided by the county midyear population. The quotients were then multiplied by 10,000, resulting in a rate of nonprofits or religious organizations per 10,000 county residents.

*Institutional confidence*. This sub-index measures commitment to society and trust in institutions [35]. Institutional confidence was assessed using 2010 census response rates and voter turnout rates from 2012 and 2016. The first indicator in the institutional confidence sub-index, 2010 census return rates, are reported by the US Census Bureau’s table “Rates for all possible geographies, including American Indian Areas” and did not require transformation [67]. Average voter rates in 2012 and 2016 were computed by dividing the number of voters in a county [68] by the county midyear population aged 18 years and older (AFF table B05003). This resulted in the rate of county residents age 18 and older participating in the 2012 and 2016 federal elections.

*Collective efficacy*. Collective efficacy was calculated using standardized violent crime rates (VCRs). VCRs tend to be a better indicator of crime rates across counties than general crime rates because they are measured and reported more consistently across jurisdictions [35]. VCRs are calculated using a three-year moving average constructed from the FBI’s UCR data via SimplyAnalytics [69]. For most years, mean county VCRs are calculated using data from several years prior to a given year. For example, 2013 VCRs were calculated using data from 2009 to 2011. This pattern follows for all years from 2013 to 2017. However, due to a delayed release, 2016 VCRs are not available. Therefore, for all counties, 2018 VCRs are the same as 2017 VCRs. Standardized VCRs were inverted so that higher scores indicate higher levels of social capital.

*Social capital data availability.* Each sub-index was constructed using the most recently available data. However, not all social capital data are from the same time. The family unity and informal civil society indicators are all available on a yearly basis. Thus, values for these indicators are the same for each month in every calendar year. The institutional confidence indicators do not change over time, so each indicator takes on a single value over the entire course of the study. The collective efficacy indicator is released semi-regularly, so collective efficacy values take on the most recent value and are assigned for each month in the given calendar year. This data collection and integration process reflects the methods established by the JEC in their Social Capital Project [35]. Following these steps ensured that the data used for this project were similar to the data gathered for the original iteration of the Social Capital Project.

#### Dependent variable: CVD mortality rate

CVD mortality rates were assembled from the CDC’s WONDER underlying cause of death data. CDC WONDER data were compiled using death certificates that identify cause of death [70]. CDC WONDER documentation indicates that, when possible, an individual’s county of residence is given on their death certificate, mitigating of the issue of migration away from the affected areas. We requested the number of deaths from diseases of the circulatory system (ICD-10 codes I00-I99, alternatively CVD mortality) for all counties affected by Hurricane Matthew for each month in every year from 2013 to 2018. Death certificates are individual-level data. We calculated county-level CVD mortality rates by dividing the number of monthly CVD deaths in a county by the county midyear population (AFF table B01003) and multiplying the quotient by 10,000. This method follows previous methods for calculating measures of mortality for populations in small geographic units after hurricanes when age-specific data are not available [71]. Due to data censorship rules, research on post-disaster health outcomes in small geographic units often relies on crude mortality rates [70, 72–74].

#### Control variables: county-level sociodemographics

Sociodemographic information were pulled from the Census Bureau’s AFF tables DP05, S1902, and DP02 [65]. All data use AFF five-year estimates. Compared to one-year estimates, five-year estimates better reflect overall county trends, have substantially fewer missing data, and are ideal for measuring small geographic units [65]. Sociodemographic variables have known associations with vulnerability to environmental hazards [75].

Sociodemographic variables included the percentage of a county over age 65 and the percentage of county residents with a bachelor’s degree. The log of average county income, percent of a county that is black, or Hispanic are reported as raw numbers and are transformed. The log of mean county income was computed by taking the natural log of each county’s average income. Percent of a county that is black, or Hispanic was constructed by dividing the number of black or Hispanic county residents by the midyear population in each county.

### Analytic strategy

**Fig. 4:**
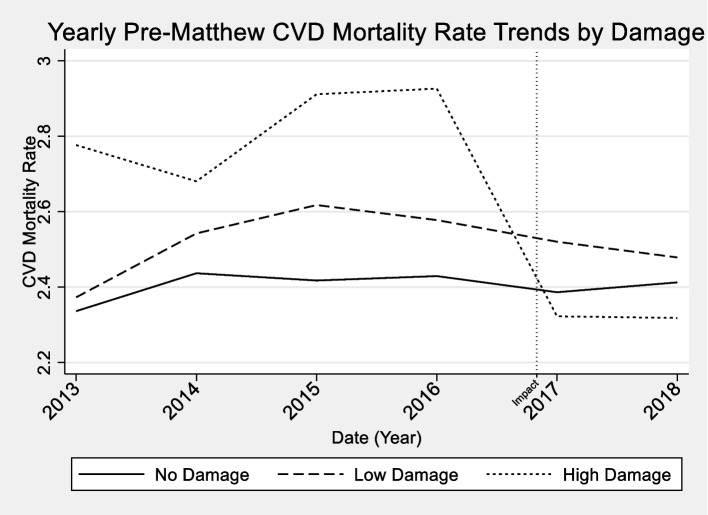
Yearly Trends in Average CVD Mortality Rates In Counties Affected by Hurricane Matthew

Our data were at the county level, so we used two population-level techniques to model the data. First, we used regression adjustment (RA) models using a robust Poisson regression to estimate the average treatment effect of the treated (ATT) of CVD mortality rates in counties affected by hurricane Matthew. We estimated the ATT in models with no social capital variables or sociodemographic controls. These are followed by models with social capital indicators. Full models include social capital indicators and adjust for county level sociodemographic characteristics. All models were adjusted for seasonality in CVD trends using a series of dummy variables to indicate month (results available upon request). We were primarily interested in the effect of damage caused by Hurricane Matthew on CVD mortality, using “no damage,” as a control group, and using “low-damage,” and “high-damage” as separate treatment groups. However, our data did not display parallel trends across levels of damage, so a difference-in-difference analysis was not appropriate for the data (Fig. 4) [76–78]. When data display heterogenous treatment effects and assignment to treatment groups is not random, RA models can produce unbiased estimates of the effect of treatment variables on outcomes by fitting multiple regression models for each treatment level (no damage, low-damage, high-damage) and comparing the predicted outcome at each treatment level [79–81]. RA results are conditional means for each treatment level. ATT models treat each analytic group as its own strata. Thus, ATT models represent the change in mean levels of CVD mortality compared to the counterfactual that the “treatment” (Hurricane Matthew) had never occurred. Higher levels of CVD mortality at a given level of hurricane damage, compared to the control group (no damage counties), suggests that hurricane damage is positively associated with CVD mortality.

After estimating the changes in mean CVD mortality by level of damage, we used generalized estimating equations (GEE) modeling to estimate the moderating effect of the full social capital index, and social capital sub-indices on CVD mortality in both low-damage and high-damage counties. GEEs are powerful tools for estimating population averages because they control for spatial autocorrelation in time-series [82]. All population average models and were estimated using a Poisson distribution, control for clustering at the county level, and control for temporality using a series of month level dummy variables [34, 83]. GEE modeling was conducted using a stepwise approach, estimating associations among hurricane damage, social capital, and CVD mortality with and without sociodemographic control variables. For all models, RA and GEE, we used complete case analysis, as our data cannot be assumed to be missing at random [84].

This was an observational study using publicly available data, and it was deemed as “Exempt” by the University of Alabama at Birmingham Institutional Review (IRB #300004140). Data collection procedures follow guidelines from the CDC and JEC [49, 64] Study design and reporting follow all relevant Strengthening the Reporting of Observational studies in Epidemiology (STROBE) guidelines [85].

## Results

### Descriptive and bivariate statistics

We compiled information on sample sizes and variable descriptive statistics in Table 2. The full sample (January 2013 to April 2018 for all counties) had information from 183 unique counties for a total of 8893 observations. Descriptive statistics encompass county-level averages over the course of the study. Of the 183 counties being studied, approximately 69%, 126 counties, were in the “no damage” sample. About 7% of the sample (12 counites total) experienced low levels of hurricane damage; 25% (45 counties total) experienced high levels of hurricane damage. The mean county monthly CVD mortality rate was 2.50 deaths per 10,000 county residents. Counties had, on average, mean levels of total social capital. Positive family unity and informal civil society scores of 0.51 and 0.41, respectively, indicate that counties tended to have above average levels of these forms of social capital. However, below average institutional confidence scores, − 0.40, indicate that counties in the sample tended to have lower than average census response rates and voter turnout. A mean score of .21 on the collective efficacy measure indicates that most counties in the sample had average levels of violent crime. For counties in the sample, an average of 16.5% of the county was age 65 or older, 19.8% of the county was black, 9.7% of the county identified as Hispanic, and 27.5% of county residents held at least a bachelor’s degree. (For Spearman’s correlation coefficients see: SM, Table 1, summary of results in Table S1).

**Table 2:** Comparison of All Variable Means Between No, Low, and High Damage Counties (n = 183)

	Mean No Damage *(n = 126)*	Mean Low Damage *(n = 12)*	Mean High Damage *(n = 45)*
Monthly CVD Mortality Rate*	2.50	2.50	2.46
Social Capital Index^†^	0.18	−1.13 ˡ	0.17
Family Unity^†^	0.54	−0.01 ˡ	0.16 ˡ
Rate of Single Parent Households	33.38	33.92	34.80 ˡ
Rate of Unmarried Births	38.61	43.77 ˡ	39.31
Rate of Married Women	60.54	58.98 ˡ	58.80 ˡ
Informal Civil Society^†^	0.35	−1.37 ˡ	0.38
Rate of Nonprofits*	2.53	2.35	2.80 ˡ
Rate of Religious Orgs*	7.63	4.65 ˡ	6.79 ˡ
Institutional Confidence^†^	−0.36	−0.08 ˡ	−0.47
Percent 2010 Census Response	0.76	0.75 ˡ	0.75 ˡ
Percent Voting in 2012	0.59	0.58 ˡ	0.62 ˡ
Percent Voting in 2016	0.56	0.54 ˡ	0.58 ˡ
Collective Efficacy ^†^	0.18	0.52 ˡ	0.47 ˡ
Violent Crime Rate	165.02	85.00 ˡ	97.96 ˡ
Percent Age 65+	16.40	18.50 ˡ	16.98 ˡ
Percent Black	19.67	19.27	22.31 ˡ
Percent Hispanic	9.46	20.90 ˡ	8.35 ˡ
Percent with Bachelor’s Degree	27.31	27.57	31.16 ˡ
Log Mean Income	10.81	10.88 ˡ	10.86 ˡ

*Rate per 10,000

*ˡ Mean is significantly different from “no damage” mean (p ≤ .05)*

*†Standardized composite indices*

T-tests showed significant differences between counties across CVD mortality rates, several social capital indices, and control variables (Table 3). On average compared to no damage counties, neither low-damage nor high-damage counties experienced significantly different CVD mortality rates. Low damage counties have lower scores on the full social capital index (*p* ≤ .001), family unity (*p* ≤ .001), and informal civil society (p ≤ .001) indices, and higher scores on the institutional confidence (*p* ≤ .001) and collective efficacy (*p* ≤ .001) indices. Highly damaged counties had similar levels of overall social capital and informal civil society scores as no damage counties, but lower mean social family unity (*p* ≤ .01) and institutional confidence (*p* ≤ .05) scores. Highly damaged counties had higher mean collective efficacy scores than no damage counties (*p* ≤ .001). With regard to control variables, both low and high-damage counties had a higher log mean income (p ≤ .001), and a higher percentage of residents 65 and older, on average, compared to no damage counties (p ≤ .001). High damage counties had a greater percentage of black residents (p ≤ .001) and higher percentages of the population with bachelor’s degrees (p ≤ .001), but a lower percentage of Hispanic residents (p ≤ .001) than no damage counties. Low damage counties, meanwhile, had a higher percentage of Hispanic residents than no damage counties (p ≤ .001) but were not significantly different from no damage counties with respect to the percentage of black residents, or percentage of residents with a bachelor’s degree.

**Table 3:** Descriptive Statistics for Counties in the Study Sample ( n = 183)

	Mean /% ±	SD	50th	(25th,	75th)	IQR
Percentile						
**Independent Variable**						
No Damage *(n = 126)*	68.85%					
Low Damage *(n = 12)*	6.56%					
High Damage *(n = 45)*	24.59%					
**Dependent Variable**						
Monthly CVD Mortality Rate*	2.50	1.04	2.34	(1.79,3.02)		1.23
**Moderating Variable(s)**						
Social Capital Index^†^	0.14	2.04	0.07	(−1.41, 1.50)		2.92
Family Unity^†^	0.51	2.45	0.52	(−0.89, 2.09)		0.51
Rate of Single Parent Households	33.47	8.90	33.00	(28.00, 38.00)		33.47
Rate of Unmarried Births	38.79	12.94	38.70	(29.80, 45.70)		38.79
Rate of Married Women	60.41	8.19	60.30	(55.40, 65.70)		60.41
Informal Civil Society^†^	0.40	1.38	0.43	(−0.40, 1.28)		0.40
Rate of Nonprofits*	2.54	1.55	2.10	(1.53, 3.15)		2.54
Rate of Religious Orgs Organizations*	7.50	2.89	7.06	(5.23, 9.31)		7.50
Institutional Confidence ^†^	−0.40	1.24	−0.47	(−1.40, 0.43)		−0.40
Proportion 2010 Census Response	0.76	0.04	0.76	(0.73, 0.79)		0.76
Proportion Voting in 2012	0.59	0.08	0.59	(0.55, 0.64)		0.59
Proportion Voting in 2016	0.56	0.07	0.57	(0.52, 0.61)		0.56
Collective Efficacy^†^	0.21	0.54	0.35	(−0.04, 0.60)		0.21
Violent Crime Rate	159.20	126.55	126.178	(67.58, 216.53)		159.20
**Control Variables**						
Percent Age 65+	16.49	5.89	15.33	(12.21, 19.32)		7.11
Percent Black	19.80	14.76	16.37	(8.56, 27.24)		18.68
Percent Hispanic	9.73	8.87	7.01	(4.90, 11.58)		6.68
Percent with Bachelor’s Degree	27.52	10.82	25.28	(19.55, 33.63)		14.08
Log Mean Income	10.81	0.25	10.78	(10.65, 10.94)		0.29

*Rate per 10,000

^†^Standardized composite indices

### Regression adjustment results

Regression adjustment results provide estimates for changes in monthly CVD mortality rates due to levels of hurricane damage both with, and without covariates. These results can be found in Table 4. Mean CVD mortality for no damage counties are interpreted as the baseline CVD mortality rate had Hurricane Matthew never occurred. CVD mortality means for low-damage and high-damage counties represent with change in CVD mortality rates within each damage strata as a result of Hurricane Matthew. Significance for low and high-damage CVD mortality rates indicates that mean CVD mortality is significantly different from no damage counties. CVD mortality models with controls are presented in separate columns so that the change in CVD mortality for all covariates in all models can be fully reported. β coefficients for social capital indices and control variables represent the absolute change in monthly CVD mortality rates due to a one unit change in the sub-index or control variable.

**Table 4:** Regression Adjusted Average Treatment Effect of the Treated Means for Counties (n = 183) by Level of Damage

	(1)	(2)	(3)	(4)	(5)	(6)	(7)
No Damage Mean	2.50, *0.01*	2.32, *0.02*			2.34, *0.04*		
	(2.47, 2.53)	(2.28, 2.37)			(2.27, 2.41)		
Low Damage Mean	2.50, *0.05*		2.50***, *0.05*			2.50***, *0.03*	
	(2.39, 2.62)		(2.36, 2.65)			(2.37, 2.64)	
High Damage Mean	2.44, *0.04*			2.34, *0.06*			2.51*, *0.08*
	(2.32, 2.55)			(2.18, 2.50)			(2.29, 2.74)
Family Unity^†^		−0.06***, *0.00*	−0.05*, *0.02*	−0.06***, *0.01*	−0.01***, *0.00*	0.05, *0.04*	−0.02, *0.01*
		(−0.06, −0.05)	(− 0.09, − 0.00)	(−0.08, − 0.04)	(−0.02, − 0.01)	(−0.03, 0.13)	(− 0.04, 0.00)
Informal Civil Society^†^		0.07***, *0.00*	−0.01, *0.03*	0.03, *0.03*	0.06***, *0.00*	0.09***, *0.02*	0.06***, *0.02*
		(0.06, 0.07)	(−0.06, 0.04)	(−0.02, 0.07)	(0.06, 0.07)	(0.04, 0.14)	(0.03, 0.10)
Institutional Confidence^†^		0.03***, *0.00*	0.12***, *0.02*	0.04***, *0.01*	0.02***, *0.00*	−0.10***, *0.02*	0.05***, *0.01*
		(0.03, 0.04)	(0.08, 0.16)	(0.02, 0.07)	(0.01, 0.02)	(−0.13, −0.06)	(0.03, 0.07)
Collective Efficacy^†^		−0.05***, *0.01*	0.30***, *0.05*	−0.11**, *0.04*	0.01, *0.01*	0.03, *0.03*	0.09*, *0.04*
		(−0.07, − 0.04)	(0.20, 0.40)	(− 0.19, − 0.03)	(−0.01, 0.02)	(− 0.03, 0.08)	(0.01, 0.18)
Percent County Black					0.00 *0.00*	0.01, *0.00*	−0.01**, *0.00*
					(−0.00, 0.00)	(−0.00, 0.08)	(− 0.01, − 0.01)
Percent County Hispanic					−0.01, *0.00*	0.00, *0.00*	0.00, *0.00*
					(−0.01, −0.00)	(− 0.00, 0.00)	(− 0.01, 0.00)
Percent County Age 65+					0.03***, *0.00*	0.05***, *0.00*	0.03***, *0.00*
					(0.03, 0.03)	(0.04, 0.06)	(0.02, 0.03)
Percent County with Bachelor’s Degree					−0.00***, *0.00*	−0.00, *0.00*	−0.00***, *0.00*
					(−0.01, − 0.00)	(− 0.00, − 0.00)	(−0.00, − 0.00)
Log Mean Income					−0.17***, *0.03*	0.71*, *0.31*	−0.38**, *0.14*
					(−0.22, − 0.12)	(0.10. 1.31)	(− 0.64, − 0.10)

In models without controls, the mean monthly CVD mortality rate in counties with no hurricane damage was 2.5 CVD (95% CI [2.5, 2.5]). Neither low nor high-damage counties were significantly different from no damage counties in these models (Table 5, column 1). After controlling for social capital indices, mean monthly CVD mortality in no damage counties decreased to 2.3 (95% CI [2.3, 2.4]) deaths per 10,000 (Table 5, column 2) and the mean monthly CVD mortality in low-damage counties stayed at 2.5 (*p* ≤ .001, 95% CI [2.4, 2.6]) CVD deaths per 10,000 (Table 5, column 3). The relationship between high levels of damage and CVD mortality was not significant in these models. In full models, the mean monthly CVD mortality in no damage counties was 2.3 (95% CI [2.3, 2.6]) CVD deaths per 10,000 (Table 5, column 5). In low-damage counties monthly CVD deaths per 10,000 remained constant at 2.5 (*p* ≤ .001, 95% CI [2.4, 2.6]) CVD deaths per 10,000 (Table 5, column 6), while Monthly CVD mortality per 10,000 increased to 2.5 (*p* < .05, 95% CI [2,3, 2.7]) in high-damage counties (Table 5, column 7). A 0.17% increase in CVD mortality suggests that if the most populous county in the high-damage sample, Wake County, NC (2016 population midyear 998,576) would experience about 17 additional CVD mortality deaths per month for the 18 months following Hurricane Matthew,

**Table 5:** Moderating Effects of Social Capital Indices on CVD Mortality in Low Damage Counties (n = 12)

	(1)	(2)	(3)	(4)	(5)	(6)
Month (Time)	1.00**, *0.00*	1.00, *0.00*	1.00, *0.00*	1.00, *0.00*	1.00, *0.00*	1.00, *0.00*
	(1.00, 1.00)	(0.99, 1.00)	(0.99, 1.00)	(0.99, 1.00)	(0.99, 1.00)	(0.99, 1.00)
Social Capital Index^*†*^	0.94, *0.14*	0.97, *0.13*				
	(0.70, 1.26)	(0.74, 1.25)				
Month*Social Capital Index^*†*^	1.00, *0.00*	1.00, *0.00*				
	(0.99, 1.00)	(0.99, 1.00)				
Family Unity^*†*^			1.06, *0.29*	1.00, *0.02*	1.01, *0.01*	1.00, *0.02*
			(0.62, 1.79)	(0.97, 1.04)	(0.98, 1.04)	(0.97, 1.03)
Informal Civil Society^*†*^			1.00, *0.02*	0.95, *0.16*	0.99, *0.02*	1.01, *0.02*
			(0.97, 1.04)	(0.68, 1.32)	(0.95, 1.03)	(0.95, 1.06)
Institutional Confidence^*†*^			0.91***, *0.02*	0.91***, *0.02*	1.15, *0.16*	0.91***, *0.02*
			(0.87, 0.95)	(0.87, 0.95)	(0.88, 1.50)	(0.87, 0.94)
Collective Efficacy^*†*^			1.00, *0.02*	1.00, *0.02*	0.99, *0.02*	1.10, *0.41*
			(0.96, 1.04)	(0.96, 1.04)	(0.95, 1.03)	(0.52, 2.30)
Month*Family Unity^*†*^			1.00, *0.00*			
			(0.99, 1.00)			
Month*Informal Civil Society^*†*^				1.00, *0.00*		
				(0.99, 1.00)		
Month*Institutional Confidence^*†*^					1.00, *0.00*	
					(0.99, 1.00)	
Month*Collective Efficacy^*†*^						1.00, *0.00*
						(0.99, 1.00)
Percent County black		1.00, *0.01*	1.00, *0.00*	1.00, *0.00*	1.00, *0.00*	1.00, *0.00*
		(0.99, 1.01)	(0.99, 1.01)	(0.99, 1.01)	(0.99, 1.01)	(0.99, 1.01)
Percent County Hispanic		1.00, *0.00*	0.99***, *0.00*	0.99**, *0.00*	0.99***, *0.00*	0.99***, *0.00*
		(0.99, 1.01)	(0.99, 0.99)	(0.99, 0.99)	(0.99, 0.99)	(0.99, 0.99)
Percent County with Bachelor's Degree		0.99***, *0.00*	0.99*, *0.01*	0.99*, *0.01*	0.99*, *0.01*	0.99*, *0.01*
		(0.98, 0.99)	(0.98, 0.99)	(0.98, 0.99)	(0.98, 0.99)	(0.98, 0.99)
Percent County Age 65+		1.03***, *0.01*	1.04***, *0.01*	1.04***, *0.01*	1.04***, *0.01*	1.04***, *0.01*
		(1.02, 1.05)	(1.03, 1.05)	(1.02, 1.05)	(1.03, 1.06)	(1.03, 1.05)
Log Mean Income		0.77, *0.15*	1.04, *0.25*	1.08, *0.26*	1.03, *0.25*	1.07, *0.30*
		(0.54, 1.12)	(0.64, 1.65)	(0.66, 1.74)	(0.54, 1.12)	(0.62, 1.85)
*Total Observations = 768*						

*Estimates in the table are:* IRR*, SE (95% Confidence Interval)*

^*†*^*Standardized composite indices*

*** p < .001, ** p < .01, * p < .05

*(1) Moderating effect of social capital index, no controls (2) Moderating effect of social capital index net of controls*

*(3) Moderating effect of family unity net of controls (4) Moderating effect of informal civil society net of controls*

*(5) Moderating effect of institutional confidence net of controls (6) Moderating effect of collective efficacy net of controls*

In general, social capital sub-indices displayed a weak and inconsistent effect on CVD mortality after controlling for sociodemographic factors. A standard deviation (SD) change in the informal civil society sub-index was associated with increased CVD mortality in all counties, regardless of level of damage (Table 5, columns 5-7), with the strongest effect in low-damage counties (no damage β = 0.06 *p* ≤ .001, 95% CI [0.06, 0.07]; low-damage β = 0.09, p ≤ .001, 95% CI [0.04, 0.14]; high-damage β = 0.6, p ≤ .001, 95% CI [0.03, 0.10]). A SD change in collective efficacy was associated with similar increases in CVD mortality in high-damage counties only (β = 0.09, p ≤ .001, 95% CI [0.01, 0.18]). A SD change in family unity was associated with a modest but significant reduction (β = − 0.01, p ≤ .001, 95% CI [− 0.02, − 0.01]) in CVD mortality among no damage counties, and no change in low-damage or high-damage counties. Finally, a SD change in institutional confidence was associated with increased CVD mortality in no damage (β = 0.02, p ≤ .001, 95% CI [0.01, 0.02]) and high-damage (β = 0.05, p ≤ .001, 95% CI [0.03, 0.07]) counties, but reduced CVD mortality in low-damage counties (β = − 0.10, p ≤ .001, 95% CI [− 0.13, − 0.06]).

In full models, sociodemographic controls tended to run in expected directions (Table 5, columns 5-7) with two major exceptions. For high-damage counties each percentage increase in black county residents was associated with a decrease in CVD mortality rates by 0.01 (*p* < .01, 95% CI [− 0.01, − 0.01]) (Table 5, columns 5,7). The second result worth noting is the association between log mean income and CVD mortality in low-damage counties. In low-damage counties, each unit increase in the log of mean income was associated with an increase in monthly CVD mortality rates by 0.71% (*p* ≤ .05, 95% CI [0.10, 1.31]). While the magnitude of this effect was not surprising, the direction of the effect was unexpected. The association was as expected in no damage and high-damage counties.

### Population average results

Estimates for GEE population average models examining the moderating relationship between social capital and CVD mortality for low-damage counties are presented in Table 5. Table 6 presents these same estimates for high-damage counties. Neither the full social capital index, nor any of the social capital sub-indices, were consistently associated with reduced CVD mortality trends in either low or high-damage counties. However, in low-damage counties, a SD increase in the institutional confidence was consistently associated with decreased CVD mortality rates immediately after Hurricane Matthew (IRR = 0.91, *p* ≤ .001, 95% CI [0.87, 0.95]) (Table 5, columns 3,4). The exception to this finding was when testing the moderating effect of institutional confidence and time on monthly CVD mortality rates (Table 5, column 5). For both sets of counties, control variables ran in the expected directions, but the relationships were generally null. In low-damage counties the percentage of county that is Hispanic (IRR = 0.99, p ≤ .001, 95% CI [0.99, 0.99]; Table 5, columns 3-6) and the percentage of a county that holds a bachelor’s degree (IRR = 0.99, *p* < .05, 95% CI [0.98, 0.99]; Table 5, columns 3-6) both showed slightly protective effects. In these same counties, the percentage of a county aged 65 and older was associated with increased CVD mortality rates (IRR = 1.04, p ≤ .001, 95% CI [1.03, 1.05]; Table 5, columns 3,5,6). In high-damage counties, the only significant relationship was for the log of mean income (IRR = 0.62, 95% CI [0.42, 0.91], p < .05; Table 6, column 6), indicating an overall protective effect.

**Table 6:** Moderating Effects of Social Capital Indices on CVD Mortality in High Damage Counties (n = 45)

	(1)	(2)	(3)	(4)	(5)	(6)
Month (Time)	1.00***, *0.00*	1.00, *0.00*	1.00, *0.00*	1.00, *0.00*	1.00, *0.00*	1.00, *0.00*
	(1.00, 1.00)	(0.99, 1.00)	(0.99, 1.00)	(0.99, 1.00)	(0.99, 1.00)	(0.99, 1.00)
Social Capital Index^*†*^	0.99, *0.15*	1.01, *0.17*				
	(0.73, 1.34)	(0.73, 1.40)				
Month*Social Capital Index^*†*^	1.00, *0.00*	1.00, *0.00*				
	(0.99, 1.00)	(0.99, 1.00)				
Family Unity^*†*^			0.90, *0.10*	0.98, *0.02*	0.98, *0.02*	0.98, *0.01*
			(0.72, 1.13)	(0.95, 1.01)	(0.95, 1.01)	(0.95, 1.01)
Informal Civil Society^*†*^			1.06, *0.05*	1.06, *0.04*	1.13, *0.22*	1.06, *0.04*
			(0.95, 1.01)	(0.98, 2.09)	(0.97, 1.07)	(0.97, 1.07)
Institutional Confidence^*†*^			1.02, *0.03*	1.43, *0.28*	1.02. *0.03*	1.01, *0.03*
			(0.97, 1.15)	(0.97, 1.14)	(0.77, 1.65)	(0.97, 1.15)
Collective Efficacy^*†*^			0.97, *0.03*	0.96, *0.03*	0.98, 0.03	0.60, 0.27
			(0.91, 1.03)	(0.91, 1.02)	(0.92, 1.04)	
Month*Family Unity^*†*^			1.00, *0.00*			
			(0.99, 1.00)			
Month*Informal Civil Society^*†*^				1.00, *0.00*		
				(0.99, 1.00)		
Month*Institutional Confidence^*†*^					1.00, *0.00*	
					(0.99, 1.00)	
Month*Collective Efficacy^*†*^						1.00, *0.00*
						(0.99, 1.00)
Percent County black		1.00, *0.00*	1.00, *0.00*	1.00, *0.00*	1.00, *0.00*	1.00, *0.00*
		(0.99, 1.01)	(0.99, 1.01)	(0.99, 1.01)	(0.99, 1.01)	(0.99, 1.01)
Percent County Hispanic		0.99, *0.01*	0.99, *0.01*	0.99, *0.01*	0.99, *0.01*	0.99, *0.01*
		(0.97, 1.01)	(0.97, 1.01)	(0.97, 1.01)	(0.98, 1.01)	(0.97, 1.01)
Percent County Bachelor's Degree		0.99, *0.00*	0.99, *0.00*	0.99, *0.00*	0.99, *0.00*	0.99, *0.00*
		(0.99, 1.01)	(0.99, 1.00)	(0.99, 1.00)	(0.99, 1.00)	(0.99, 1.00)
Bachelor’s Degree		(0.99, 1.01)	(0.99, 1.00)	(0.99, 1.00)	(0.99, 1.00)	(0.99, 1.00)
Percent County Age 65+		1.02, *0.01*	1.01, *0.01*	1.02, *0.01*	1.02, *0.01*	1.02, *0.01*
		(0.97, 1.05)	(0.99, 1.04)	(0.99, 1.04)	(0.99, 1.04)	(0.99, 1.04)
Log Mean Income		0.66*, *0.14*	0.61*, *0.13*	0.65**, *0.11*	0.64*, *0.13*	0.62*, *0.12*
		(0.43, 0.99)	(0.40, 0.93)	(0.47, 0.90)	(0.43, 0.95)	(0.42, 0.91)
*Total Observations 2,208*						

*Estimates in the table are:* IRR*, SE (95% Confidence Interval)*

^*†*^*Standardized composite indices*

*** p < .001, ** p < .01, * p < .05

*(1) Moderating effect of social capital index, no controls (3) Moderating effect of social capital index net of controls*

*(3) Moderating effect of family unity net of controls (4) Moderating effect of informal civil society net of controls*

*(5) Moderating effect of institutional confidence net of controls (6) Moderating effect of collective efficacy net of controls*

## Discussion and conclusion

This study examined if hurricane damage is associated with increased CVD mortality rates in high and low-damage counties compared to counties that were undamaged, and if social capital reduces CVD mortality. We found that, net of social capital indicators and sociodemographic controls, both low-damage and high-damage counties experienced higher levels of CVD mortality after Hurricane Matthew than counties that experienced no damage. Institutional confidence was associated with reduced CVD mortality immediately following Hurricane Matthew in low-damage counties, but no measure of social capital was associated with reduced CVD mortality trajectories after Hurricane Matthew. These findings suggest that the effects of hurricane damage exacerbate CVD mortality, but that effects of social capital on CVD mortality in post-hurricane settings may be limited to areas with lower levels of hurricane damage.

Our findings are consistent with previous literature on hurricanes and CVD mortality, which suggest that CVD events occur at an elevated rate after major hurricanes [3, 14, 15, 36]. It is likely that the emotional and physical stressors caused by Hurricane Matthew, combined with interruptions in normal routines and coping resources exacerbate conditions associated with CVD mortality [86–90]. Among social capital variables, only the institutional confidence sub-index was associated with significantly reduced CVD mortality rates, and only in low-damage counties. This finding is consistent with other social capital and health in disaster research [33]. Areas with high levels of institutional confidence ought to be more likely to follow the advice of public health messaging, may be better equipped to know what to do after hurricanes, and may have an easier time advocating for themselves in the post-disaster process [37, 91]. Why there was no long-term protection from institutional confidence is unclear. Both findings, immediate protective effect and null long-term effect, warrant further investigation. It is also worth noting that among the RA models, the informal civil society sub-index showed consistent positive associations with CVD mortality rates in low-damage counties. This may indicate that the informal civil society may isolate individuals or transmit negative health behaviors after hurricanes, in line with the “dark side,” or “Janus-faced nature,” of social capital [42, 46].

Our findings point towards social factors, specifically age and income distribution in the community, as important factors for CVD mortality after hurricanes. Higher levels of income, wealth, and education have been consistently associated with a decreased risk of all-cause and CVD mortality, as well as improved health outcomes in hurricane survivors [92–96]. Communities with a high number of elderly residents may be at particularly high risk for elevated CVD mortality in the wake of hurricanes. This finding may be driven by the relationship between age and CVD mortality, but may also be related to social isolation and a lack of mobility associated with aging [75, 97, 98]. Communities with a high proportion of residents aged 65+ may do well to make sure that elderly citizens are able to evacuate if necessary and are able to access food, water, and medical resources after hurricanes.

### Limitations

There are several limitations to the study. First, there are data capture issues. The CDC WONDER system redacts data from counties when raw CVD mortality is below ten people under specific sets of parameters. Since complete case analysis is used, data are more likely to be from counties with larger populations. In a similar vein, age-adjusted mortality rates are not available. Data for social capital, even though they are not redacted, are also limited. Most social capital indicators are available on an annual basis, but data for some indicators are available for only a single time point throughout the study. For this the current study, monthly data collected before and after hurricanes would be ideal. It is possible that a different social capital measurement strategy could pick up associations in the current study or indicator that was collected more consistently, might show effects on CVD mortality trends not seen here. Data limitations notwithstanding, our research provides evidence for the relationship between high levels of hurricane damage and CVD mortality after Hurricane Matthew.

Our social capital measure is one of many possible ways to measure social capital. Alternative measures, such as a resource generator, [25] may be helpful in highlighting the specific resources that improve CVD outcomes after hurricanes. Alternative community-level measures, such as Putnam’s social capital index, [99] have also been used to understand how community level resources can be leveraged to improve health outcomes. However, Putnam’s index faces shortcomings in terms of data availability [100]. There have been attempts to improve on Putnam’s social capital index, but there is no universally agreed upon operationalization of the concept. More recently the Penn State Social Capital Index [56] has been proposed as a more definitive county-level social capital measure. However, a lack of data updates and some questions over its validity [101] have left researchers looking for new county-level indicators that are up-to-date, representative, and inexpensive to compile.

The JEC social capital index offers some fixes by utilizing publicly available, current data to construct a measure that reflects the place-based relational, behavioral, structural, and cognitive elements of social capital. The JEC index has been validated against other measures, performs well, and has been used to advance various health outcomes research in the United States, [58, 102–105] but not post-hurricane CVD trends in the socially and epidemiologically disadvantaged South [77].

Although the JEC social capital measure is useful, the index has its own shortcomings. One of the contributions of our study is highlighting limited utility of the family unity index, which uses a limited definition of family and may be politically biased. In the 2019 US Census, 6.6% of households had a single parent, 39% of women aged 35-44 were unmarried, and 33.7% of women aged 15-50 years that gave birth were unmarried [65]. The JEC-based family unity measure is restricted to nuclear-family ties and is biased in favor of two-parent childrearing households, reflecting conservative political leaning. The measurement gap is reflected in the JEC validation studies, where the family unity sub-index scores the lowest compared to other sub-indices ([35]; Table 6). While close others and family ties are critical in disaster recovery, the current JEC measure fails to fully capture the diverse nature of family relationships in the United States. Ideally, family unity would reflect diverse interpersonal relationships that generate social capital. A better measure would represent the full spectrum or diversity of family ties that help to form social capital and be supported with empirical data. This would require improved in family measures in the Census/American Community Survey, allowing for an expanded definition of family, per other research [106].

### Implications

Our research provides a foundation for future research that intends to examine the complex relationship between social capital and disaster recovery in small geographic areas using concrete, repeatable, theoretically grounded measures. Further research is needed to disentangle the specific mechanisms through which social capital operates to protect and, sometimes, harm health after disasters, both in general and in different populations. Specifically, future research could examine how social capital moderates hurricane damage to affect health outcomes in populations that may be more vulnerable to natural hazards due to socioeconomic status, race and ethnicity, or urbanicity. It is unlikely that social capital, and its constituent indices, have a uniform effect for all populations across all outcomes. Additional research can identify the circumstances and populations that are helped, and potentially harmed, by social capital. Doing so will allow researchers to provide valuable insights and perspectives for public health agencies, community leaders, and researchers as severe hurricanes become more common and increase the burden of CVD and other non-communicable diseases.

## Supplementary Information

**Additional file 1 **: **Supplementary Table S1.** Spearman's Correlation Coefficients for Social Capital Indicators. **Supplementary Figure S1.** Distribution of Informal Civil Society Coefficient. **Supplementary Figure S2.** Distribution of Confidence in Institutions Coefficient.

## Data Availability

The datasets used and analyzed during the current study are available from the corresponding author on reasonable request. Links to Publicly Available Databases: FEMA Disaster Declarations: FL: https://www.fema.gov/disaster/4283/designated-areas GA: https://www.fema.gov/disaster/4284/designated-areas SC: https://www.fema.gov/disaster/4286/designated-areas NC: https://www.fema.gov/disaster/4285/designated-areas VA: https://www.fema.gov/disaster/4291/designated-areas CDC WONDER: https://wonder.cdc.gov/ucd-icd10.html American Fact Finder: Table B01003: https://data.census.gov/all/tables?q=B01003 Table S1301 https://data.census.gov/table?q=S1301&tid=ACSST1Y2021.S1301 Table B12002 https://data.census.gov/table?q=B12002 Table B09002 https://data.census.gov/table?q=B09002 Table B05003 https://data.census.gov/table?q=B05003 Table DP05: https://data.census.gov/table?q=DP05 Table S1902 https://data.census.gov/table?q=DPS1902 Table DP02: https://data.census.gov/table?q=DP02 American Fact Finder Community Business Patterns: https://www.census.gov/programs-surveys/cbp/data/tables.html North American Industry Classification System: https://www.census.gov/naics/?58967?yearbck=2017 (Mail in Census Response) Rates for all possible geographies, including American Indian Areas: https://www.census.gov/data/developers/data-sets/decennial-response-rates.2010.html#list-tab-O35NPDJ8HC6M0FTZUV Election Administration and Voting Survey (EAVS): https://www.eac.gov/research-and-data/datasets-codebooks-and-surveys FBI Uniform Crime Reporting: https://www.fbi.gov/how-we-can-help-you/need-an-fbi-service-or-more-information/ucr/publications

## Abbreviations

AFF: American Fact Finder
ATT: Average Treatment Effect of the Treated
CBP: County Business Patterns
CDC: Center for Disease Control and Prevention
CVD: Cardiovascular Disease
DR: Disaster Declaration
EAVS: Election Administration Voting Survey
FBI: Federal Bureau of Investigation
GEE: General Estimating Equation
IRR: Incident Rate Ratio
JEC: United States Joint Economic Committee
RA: Regression Adjustment
SD: Standard Deviation
STROBE: Strengthening the Reporting of Observational studies in Epidemiology
WONDER: Wide-ranging ONline Data for Epidemiologic Research.
