# Supplementary material for: Differences in county-level cardiovascular disease mortality rates due to damage caused by hurricane Matthew and the moderating effect of social capital: a natural experiment
Source: BMC Public Health. 2023 Jan 9;23:60. doi: 10.1186/s12889-022-14919-7 (PMC9830798; doi:10.1186/s12889-022-14919-7)
Supplement: Supplementary file 1 — Additional file 1 : Supplementary Table S1. Spearman's Correlation Coefficients for Social Capital Indicators. Supplementary Figure S1. Distribution of Informal Civil Society Coefficient. Supplementary Figure S2. Distribution of Confidence in Institutions Coefficient. [file 12889_2022_14919_MOESM1_ESM.docx]

**Supplemental Material**

Differences in County-Level Cardiovascular Disease Mortality Rates due to Damage Caused by Hurricane Matthew and the Moderating Effects of Social Capital: A Natural Experiment

List of Supplementary Tables and Figures:

**Supplementary Table S1.** Spearman's Correlation Coefficients for Social Capital Indicators

**Supplementary Figure S1.** Distribution of Informal Civil Society Coefficient

**Supplementary Figure S2.** Distribution of Confidence in Institutions Coefficient

| **Supplemental Table S1.** Spearman’s Correlation Coefficients for Social Capital Indicators in the County Sample (n = 183) | | | | | | | | | | | | | | | |  |
| --- | --- | --- | --- | --- | --- | --- | --- | --- | --- | --- | --- | --- | --- | --- | --- | --- |
| Variables | (1) | (2) | (3) | (4) | (5) | (6) | (7) | (8) | (9) | (10) | (11) | (12) | (13) | (14) | |  |
| (1) Monthly CVD Mortality Rate | 1.00 |  |  |  |  |  |  |  |  |  |  |  |  |  |  |  |
| (2) Social Capital Index^†^ | -0.01 | 1.00 |  |  |  |  |  |  |  |  |  |  |  |  |  |  |
| (3) Family Unity^†^ | -0.34 | 0.42 | 1.00 |  |  |  |  |  |  |  |  |  |  |  |  |  |
| (4) Rate of Single Parent Households | 0.32 | -0.36 | -0.95 | 1.00 |  |  |  |  |  |  |  |  |  |  |  |  |
| (5) Rate of Unmarried Births | 0.36 | -0.44 | -0.87 | 0.76 | 1.00 |  |  |  |  |  |  |  |  |  |  |  |
| (6) Rate of Married Women | -0.25 | 0.36 | 0.91 | -0.85 | -0.66 | 1.00 |  |  |  |  |  |  |  |  |  |  |
| (7) Informal Civil Society^†^ | 0.23 | 0.78 | -0.12 | 0.16 | 0.05 | -0.14 | 1.00 |  |  |  |  |  |  |  |  |  |
| (8) Rate of Nonprofits* | -0.04 | 0.39 | 0.56 | -0.59 | -0.45 | 0.51 | 0.07 | 1.00 |  |  |  |  |  |  |  |  |
| (9) Rate of Religious Organizations* | 0.16 | 0.51 | -0.11 | 0.14 | -0.03 | -0.19 | 0.67 | -0.11 | 1.00 |  |  |  |  |  |  |  |
| (10) Institutional Confidence^†^ | 0.26 | 0.59 | -0.09 | 0.14 | 0.11 | -0.03 | 0.75 | 0.17 | 0.09 | 1.00 |  |  |  |  |  |  |
| (11) Percent 2010 Census Response | -0.04 | 0.39 | 0.47 | -0.49 | -0.42 | 0.40 | 0.12 | 0.79 | 0.07 | 0.07 | 1.00 |  |  |  |  |  |
| (12) Percent Voting in 2012 | -0.09 | 0.28 | 0.41 | -0.42 | -0.39 | 0.33 | 0.04 | 0.52 | 0.12 | -0.07 | 0.92 | 1.00 |  |  |  |  |
| (13) Percent Voting in 2016 | 0.04 | 0.33 | 0.26 | -0.27 | -0.26 | 0.20 | 0.21 | 0.49 | 0.19 | 0.10 | 0.90 | 0.95 | 1.00 |  | |  |
| (14) Collective Efficacy ^†^ | -0.19 | 0.38 | 0.27 | -0.25 | -0.29 | 0.22 | 0.01 | 0.22 | 0.01 | 0.01 | 0.23 | 0.19 | 0.15 | 1.00 | |  |
| *Rate per 10,000  ^†^Standardized composite indices | | | | | | | | | | | | | | | | |

|  |
| --- |

**Summary of results in Tables S1**

Spearman’s correlation coefficients (Table S1) suggest a mixed relationship between social capital indices and CVD mortality rates. The overall relationship between the full social capital index and CVD mortality rates was very weak and negative (-0.01). Both the relationship between family unity and CVD mortality (-0.34) and the relationship between collective efficacy and CVD mortality (-0.19) were also modest and negative. The relationships between informal civil society and CVD mortality (0.23) as well as institutional confidence and CVD mortality (0.26) were both moderate and positive. There also evidence that the unique social capital sub-indices share unique relationships with CVD mortality that run in different directions, rendering the effect of the full social capital index difficult to detect. The indicators that compose sub-indices tend to be moderately to strongly and positively correlated amongst themselves, and with the scores of their respective sub-indices, indicating that indices and indicators are measuring similar concepts.

**Supplementary Figure S1.** Distribution of Informal Civil Society Coefficient


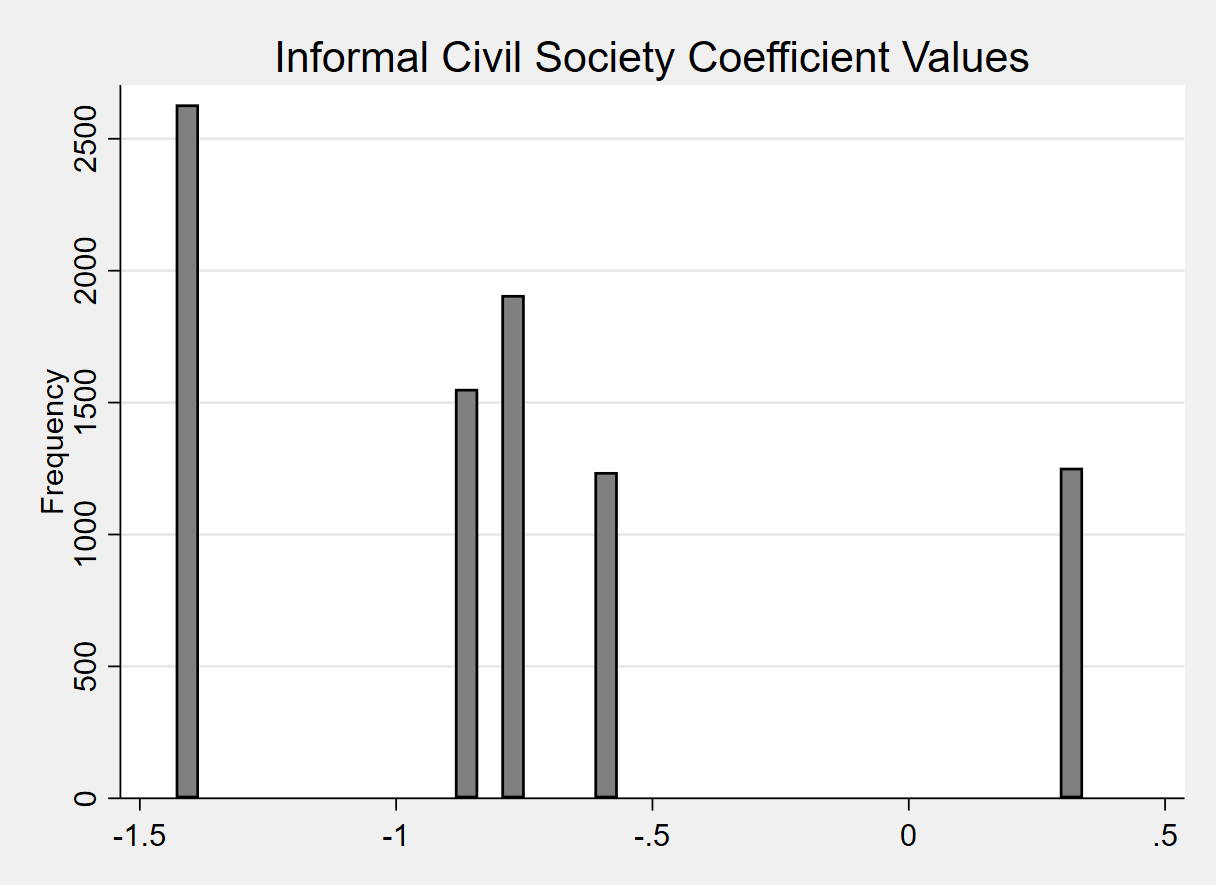


**Supplemental Figure S2.** Distribution of Confidence in Institutions Coefficient

**Summary of Figures S1-S2**

Figure S1 shows the distribution of the Informal Civil Society Index coefficient for all 106 counties over the course of the 2013-2018 study period. We excluded these data for several reasons. First, these variables were designed to be treated as continuous. However, both variables only take on 6 values over the course of the study. Second, the coefficients represent mean-standardized scores on a scale created to measure state-level social capital. For both coefficients, all values fall within 1.5 standard deviations of the mean coefficient score. The lack of variation within counties over time coupled with little variation between counties across space indicates that these variables capture a static state-level social capital variable, as opposed to the longitudinal county-level measures.

**References**

“North American Industry Classification System.” 2017. Executive Office of the President & The Office of Management and Budget. census.gov/naics.

Robert Wood Johnson. 2020. “Violent Crime Rates.” County Health Rankings and Roadmaps. 2020. https://www.countyhealthrankings.org/explore-health-rankings/measures-data-sources/county-health-rankings-model/health-factors/social-and-economic-factors/community-safety/violent-crime-rate.

United States Joint Economic Committee. 2018. “The Geography of Social Capital in America - Analysis - United States Joint Economic Committee.” April 11, 2018. https://www.jec.senate.gov/public/index.cfm/republicans/2018/4/the-geography-of-social-capital-in-america.

U.S. Census Bureau. 2018. “American FactFinder - Community Facts,” October. https://factfinder.census.gov/faces/nav/jsf/pages/community_facts.xhtml?src=bkmk.

US Census Bureau. 2019 “2010 Census Participation Rates.” Accessed September 19, 2020. https://www.census.gov/data/datasets/2010/dec/2010-participation-rates.html.

U.S. Election Assistance Commission. 2019. “Surveys and Data.” Election Administration and Voting Survey (EAVS) Datasets, Codebooks, and Survey Instruments. 2019. https://www.eac.gov/research-and-data/datasets-codebooks-and-surveys.
